# IL-17 producing mast cells contribute to synovial inflammation in non-psoriatic and psoriatic spondyloarthritis

**DOI:** 10.1186/1479-5876-9-S2-P47

**Published:** 2011-11-23

**Authors:** Troy Noordenbos, Nataliya Yeremenko, Ioana Gofita, Paul Peter Tak, Juan Cañete, Dominique Baeten

**Affiliations:** 1 Academic Medical Center/University of Amsterdam, Amsterdam, The Netherlands; 2 Hospital Clinic, Barcelona, Spain

## Background

Comparative analyses of synovitis in spondyloarthritis (SpA) versus rheumatoid arthritis (RA) suggested that the innate immune system may play a predominant role in SpA pathogenesis. Based on our recent observation of marked synovial mast cell infiltration in psoriatic arthritis (PsA), we aimed to investigate the potential contribution of mast cells to synovial inflammation in SpA.

## Methods

Synovial tissue and fluid were obtained in non-psoriatic and psoriatic SpA and RA patients. Synovial tissue was analyzed by immunohistochemistry and double immunofluorescence. Synovial fluid was analyzed by ELISA. Synovial tissue biopsies were used for ex vivo cultures.

## Results

In comparison with RA, synovial infiltration with C-kit positive mast cells was strongly and specifically increased in SpA, independently of the subtype (non-psoriatic versus psoriatic) and disease duration. Staining of mast cell granules with tryptase and toluidine blue as well as analysis of synovial fluid levels of histamine and tryptase did not indicate increased degranulation in SpA synovitis. Mast cells expressed significantly more IL-17 in SpA than RA synovitis and constituted the major IL-17 expressing cell population in SpA synovitis. Targeting mast cells with the c-kit inhibitor imatinib mesylate in ex vivo tissue cultures led to a significant decrease in the production of IL-17 as well as other pro-inflammatory cytokines. To confirm that mast cell infiltration is not a merely secondary, inflammation-dependent feature of SpA, we assessed the impact of TNF blockade on mast cell infiltration in vivo. Analysis of paired synovial biopsies before and after treatment revealed that neither the number of mast cells nor the expression of IL-17 by mast cells was affected by effective TNF blockade in SpA.

## Conclusion

The specific increase of IL-17 expressing mast cell numbers in SpA synovitis and the ex vivo targeting of these cells indicate that the mast cell/IL17 axis may contribute to the synovial inflammation in peripheral SpA. The mast cells/IL-17 axis is not affected by effective TNF blockade.

